# Influence of Meteorological Variables on Diversity and Abundance of Mosquito Vectors in Two Livestock Farms in Ibadan, Nigeria: Public Health Implications

**DOI:** 10.5376/jmr.2017.07.0009

**Published:** 2017-06-23

**Authors:** Opayele Adewale Victor, Adeniji Johnson Adekunle, Ibrahim Kolade Tahiru, Olaleye Olufemi David

**Affiliations:** 1 Department of Virology, College of Medicine, University of Ibadan, Nigeria; 2 Department of Zoology, Faculty of Science, University of Ibadan, Nigeria

**Keywords:** Arbovirus vectors, Rainfall, Temperature, Relative humidity, Nigeria

## Abstract

This study was undertaken to determine mosquito vector diversity and abundance in two livestock farms with previous history of arboviral activities in Ibadan, southwestern Nigeria. The influence of weather on mosquito populations was also studied. Adult mosquitoes were collected weekly in two proximate University of Ibadan livestock farms from March 2015 to February 2016 using CO_2_ baited CDC light trap and human landing collection methods. Mosquitoes were identified to species using morphological keys. Relationships and interaction of temperature, relative humidity, rainfall patterns and mosquito abundance were analysed using GENSTAT 4^th^ edition. Among 6,195 adult mosquitoes collected, 16 species belonging to 5 genera were morphologically identified. *Culex quinquefasciatus* constituted the most abundant mosquito, representing 46.49% of all mosquitoes encountered. High abundance in mosquito population was noted in periods succeeding months with heavy rainfall, this is when arbovirus transmission risk is highest. A positive correlation was observed between relative humidity and abundance of *Mansonia* mosquitoes. This study shows the effect of weather on natural populations of mosquito vectors. The diverse mosquito species capable of transmitting arboviruses from animal reservoirs to human and animals in livestock farms and its environment in Ibadan, Nigeria was also revealed. There is need for intensive vector control strategies targeted at reducing mosquito populations and ultimately prevention of disease outbreaks.

## Background

Mosquitoes (Diptera: Culicidae) are considered the most important arthropods of medical and veterinary importance as they rank first in the spread of many pathogens including arthropod-borne viruses (arboviruses) (Service, 2003). Over 3,500 species of mosquitoes (Harbach and Howard, 2007) are distributed throughout the tropical and temperate regions (Rajendran, 2000). Approximately 300 species of mosquitoes can transmit arboviruses with varying health implications. *Aedes* and *Culex* mosquitoes are the species most frequently associated with arbovirus transmission (Karabatsos, 1985). Arboviruses are maintained in nature through complex sylvatic, peri-domestic and urban cycles involving vectors and vertebrate host reservoirs while humans are incidental hosts (Paulo et al., 2015).

Of the over 500 known arboviruses (Karabatsos, 1985; Tsai and Chandler, 2003; Hayes et al., 2008), approximately 100 are responsible for most human/animal diseases (Karabatsos, 1985). Many arboviruses are zoonotic (Kuno, 2005). Livestock farms often serve as wildlife-livestock-human interface areas (Jones et al., 2011), as human activities interface with livestock and wildlife such as rodents, primates and birds in the search for food and water. Humans attending to livestock are exposed to vectors that maintain and transmit arboviruses and animal reservoirs that maintain these arboviruses. This scenario provides avenue for arboviruses, which are multi host infections to jump from one species to the other through shared vectors (Parrish et al., 2008).

The epidemiology of arboviruses is significantly influenced by climate. Temperature, rainfall pattern and humidity are important factors in the life-cycle of arthropod vectors, the arbovirus transmitted and the reservoirs (Rogers and Randolph, 2006; Semenza and Menne, 2009; Paz, 2015) Temperature increase causes an upsurge in the growth rates of mosquito populations, decrease the interval between blood meals, shorten incubation periods from infection to infectiousness in mosquitoes and accelerate the virus evolution rate (Baba et al., 2012). Above-average precipitation can also lead to a higher abundance of mosquitoes and increase the potential for disease outbreaks in humans (Landesman et al., 2007). A good knowledge of the changes and fluctuations that occur in natural populations of mosquito vectors is very important in the prevention and control of arboviral diseases in order to identify high and low risk transmission zones and periods (WHO, 1975).

Many arboviruses circulate in Nigeria, yellow fever for example has caused large epidemics in the past (Carey et al., 1972; Fagbami et al., 1976; Nasidi et al., 1989, there are also serologic evidence of human infection with yellow fever, Dengue, West Nile and Zika virus (Fagbami, 1979; Baba et al., 2013, Onoja et al., 2016). Antibodies to Rift Valley fever have been demonstrated in sera of humans and animals in Nigeria (Olaleye et al., 1996a; b). Antibody to WNV have also been documented in horses in Nigeria (Olaleye et al., 1989; Sule et al., 2015). There are reports that many clinical arbovirus infections have been misdiagnosed as malaria and typhoid fever in the country as well (Baba et al., 2013). All these reflect the activities of several competent mosquito vectors, capable of transmitting arboviral infections from reservoirs to other host in the country, some of these mosquito vectors have been implicated following past arbovirus epidemics (Lee and Moore, 1972; Nasidi et al., 1989; Onyido et al., 2009). Entomological surveys have documented the presence of many mosquito species in Nigeria (Boorman and Service, 1960; Okogun et al., 2005; Onyido et al., 2008; Mgbemena and Ebe, 2012). However, there are few studies that specifically target the mosquito species in livestock farms, which are wildlife-livestock-human interface areas where vectors can make contact with reservoirs before urban transmission of arboviruses occur. Knowledge of the species composition and fluctuations that takes place in mosquito vector populations in response to abiotic environmental conditions, principally weather is necessary to identify periods when these vectors thrive the most and arboviruses are at their peak of transmission.

In this study, we investigated the different mosquito species present in two livestock farms with previous history of arboviral activities. Abadina virus, Rift Valley fever virus, West Nile virus, Shamonda virus and Sabo virus have been previously isolated from animal blood samples collected from these farms in the late1960s (University of Ibadan arbovirus research project, 1970). The study also shows the influence of rainfall, temperature and relative humidity on fluctuations in the mosquito populations in the two farms.

## 1 Materials and Methods

### 1.1 Study locations

This study was carried out in Ibadan, South west Nigeria. The city has a tropical climate with two distinct seasons; a dry season from November to February characterized by low relative humidity, high environmental temperatures with low or norainfall followed by a wet/rainy season from March to October, with high relative humidity, lower environmental temperatures, abundant rainfall and often flooding (Ogolo and Adeyemi, 2009). This climatic condition favours all year round proliferation of arthropod vectors. Mosquitoes were collected from March 2015 to February 2016. The study was carried out in two livestock farms namely, the University of Ibadan dairy farm (7°27’28.6”N 3°53’57.6”E) and University of Ibadan Teaching and Research farm (07°27’14.6”N 03°53’43.4”E). They are about 2 km apart. Selection of the University of Ibadan dairy farm was based on previous documentation of establishment of vector-host arbovirus transmission cycles. The vegetation of the farms consists mainly of residual native forests and grassy areas. The vertebrate species include ruminants, reptiles, amphibians, birds and rodents. Natural water bodies are present close to the farms and water supply for animals often create artificial breeding sites where immature mosquitoes can develop. The University of Ibadan Teaching and Research farm is very close to a protected area (University of Ibadan, botanical garden) where natural forest vegetation and a variety of flowers are preserved; this park is also the home to many birds and rodents.

### 1.2 Mosquito collection and identification

Mosquitoes were collected using carbon dioxide-baited Centre for Disease Control (CDC) light traps and human landing collection (HLC) methods. HLC is the only effective method for sampling sylvatic *Aedes* and the most appropriate method for determining human risk of infection (Diallo et al., 2012). The two methods were combined in order to have representative catch of the different mosquito species present at the farms. For each site, one CDC trap was set close to the cattle shed while two trained mosquito scouts collected mosquitoes from two locations in the vegetation within the farm. Collectors were dressed in thick clothing materials with hoods, hand gloves and socks. They collected mosquitoes landing on their socks covered legs to minimise exposure to mosquito bites. The collection was done once weekly on each farm for a5 hours trapping periods from 16:00 to 21:00. The sampling pattern defined at the beginning of the collection season and taken throughout the study period and trap sites were not changed. Weather data namely rainfall, relative humidity and temperature were taken into consideration throughout the study period. This was obtained from the meteorological station in the Department of Geography, University of Ibadan. Mosquitoes that were collected were killed by freezing and stored in an icebox containing dry ice. They were then transported to the Department of Virology, University of Ibadan laboratory where they were identified using morphological keys, including Edwards (1941); Gillies and de Meillon (1968) and Harbach (1988).

### 1.3 Statistical analysis

Data on rainfall, temperature, relative humidity, and the different mosquito genera were documented using the MS Excel 2013 software and statistical analysis was done using GenStat 10.3DE software package 4^th^ Edition. Pearson’s correlation (*r*) was calculated to ascertain the relationship between various metrological variables and abundance of mosquitoes from different genera at 5% level of significance.

## 2 Results

### 2.1 Mosquito diversity and abundance

Overall, 6,195 mosquitoes belonging to five genera and 16 species were captured. The most common species was *Culex quinquefasciatus* (46.49%), followed by *Mansonia africana* (24.87%) (Table 1).

### 2.2 Influence of weather on mosquito population

**Figure 1:**
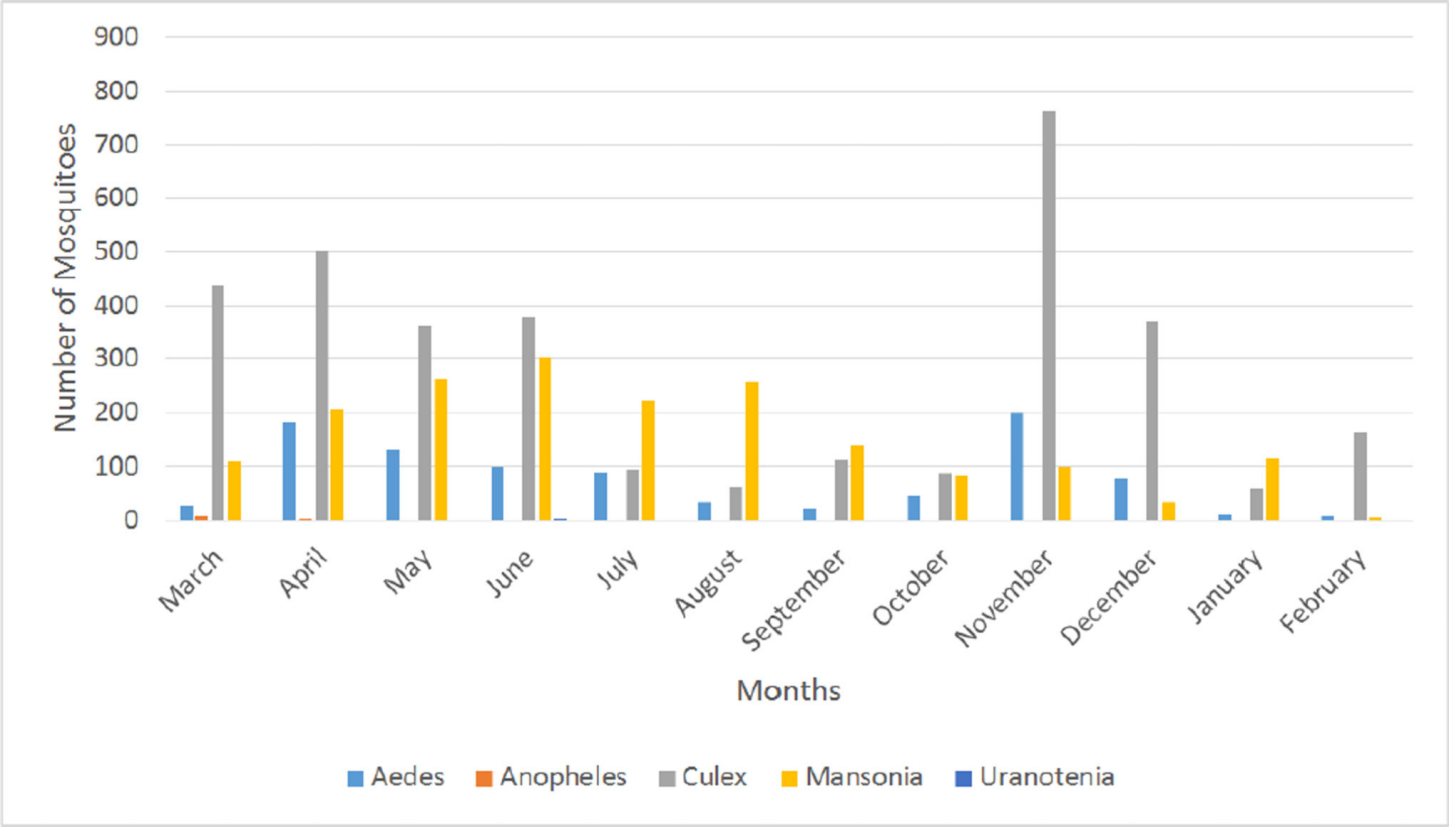
Genera of mosquitoes collected from March 2015 to February, 2016

**Figure 2:**
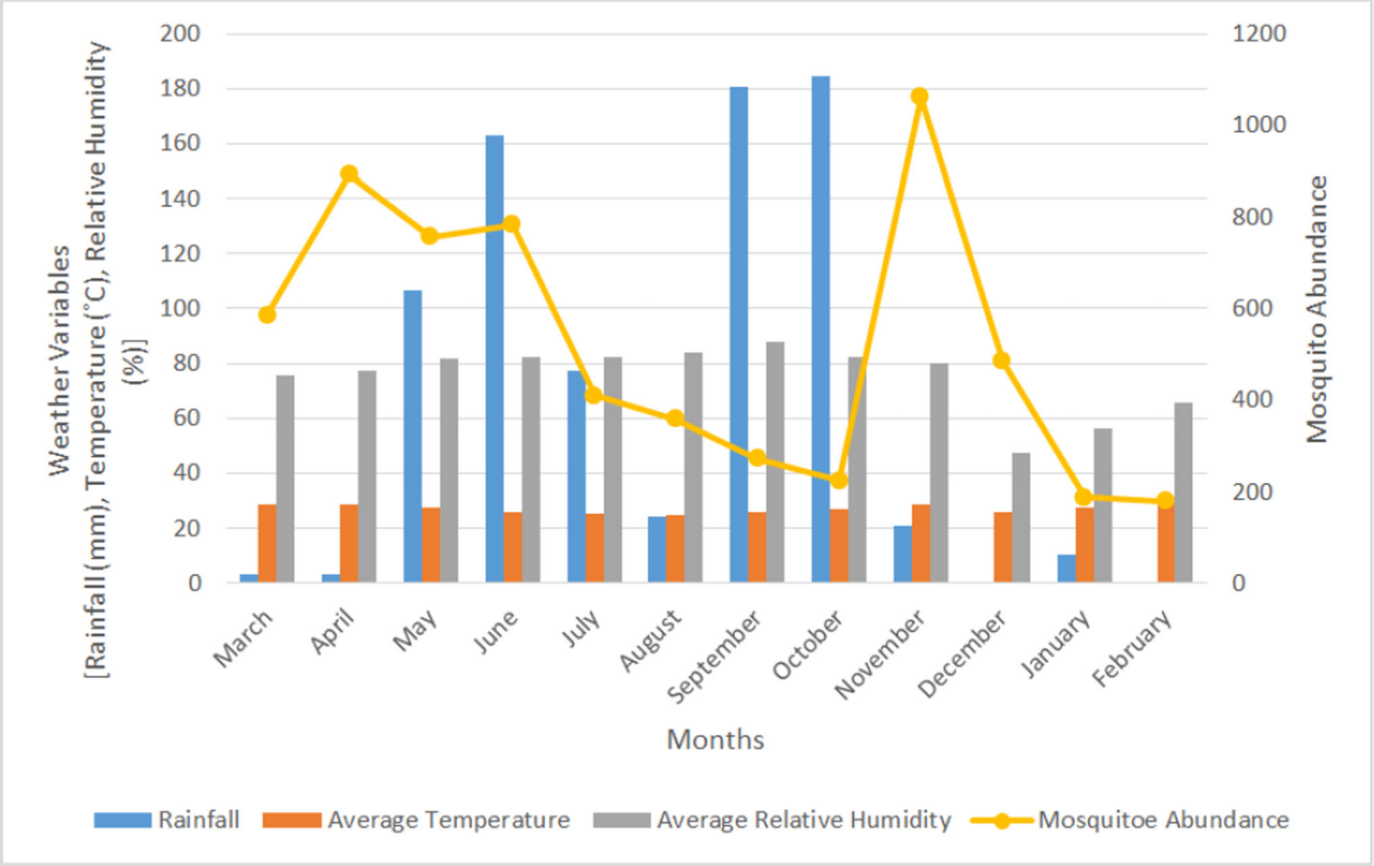
Mosquito abundance, amount of rainfall, temperature and relative humidity from March 2015 to February 2016

Populations of mosquitoes in different genera fluctuated during the study period (Figure 1). Total mosquito abundance ranged from 179 to 1062. The monthly quantities of rainfall ranged from 0.00 mm to 184.30 mm, while the mean monthly environmental temperature ranged from 24.96°C to 29.24°C. The mean monthly relative humidity ranged from 47.33% to 88.00% (Figure 2). A positive correlation (r = 0.60, *P* = 0.04) was observed between relative humidity and abundance of *Mansonia* mosquitoes.

**Figure 3:**
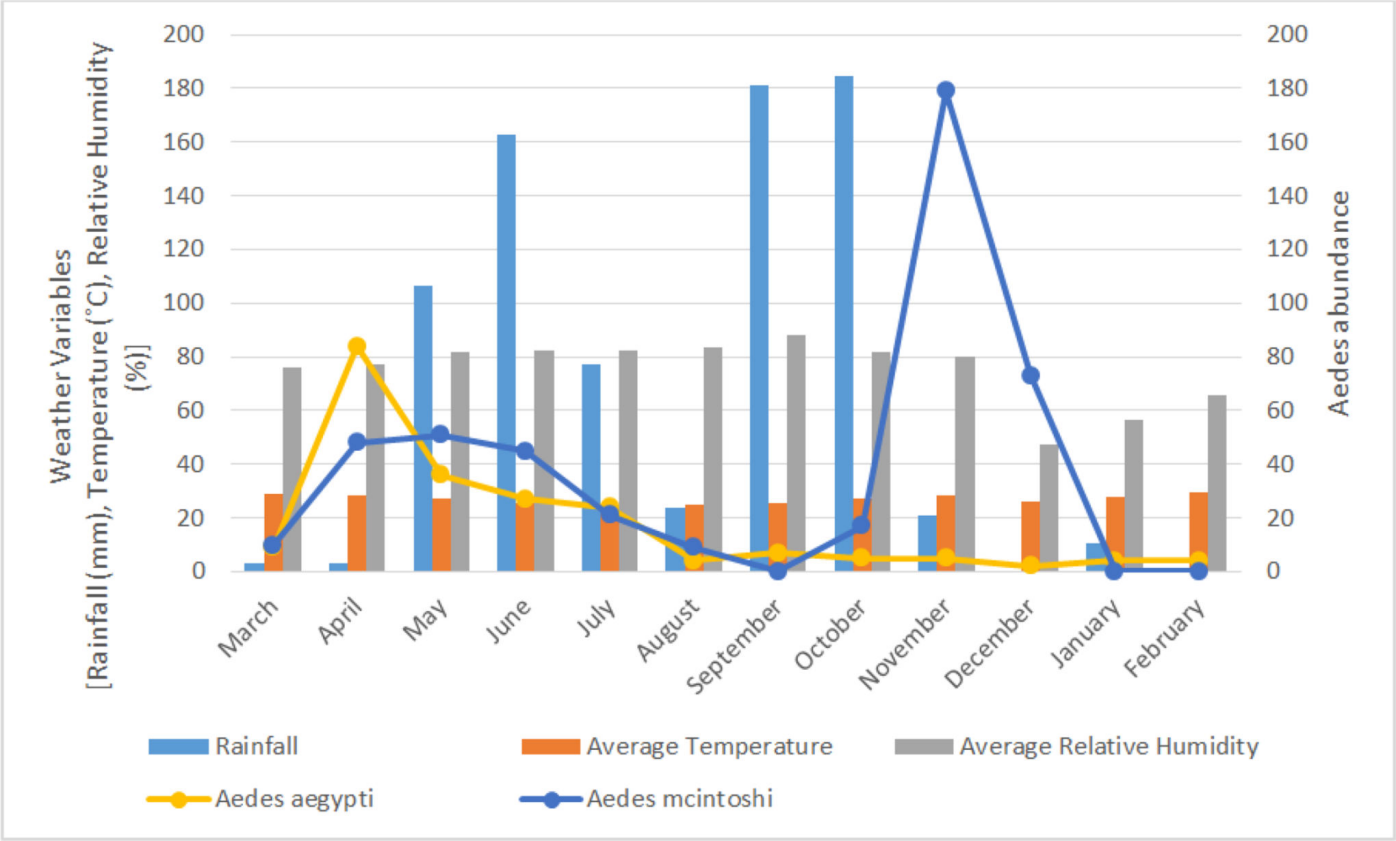
Two most occurring *Aedes* mosquitoes, amount of rainfall, temperature and relative humidity from March 2015 to February 2016

*Aedes aegypti* and *Ae. mcintoshi* were the two most occurring *Aedes* mosquitoes, population of *Ae. aegypti* was higher during the rainy season compared to the dry season. The population of *Ae. mcintoshi* declined towards the end of the rains. However, a sharp rise in population was observed immediately after the heavy rains subsided (Figure 3).

**Figure 4:**
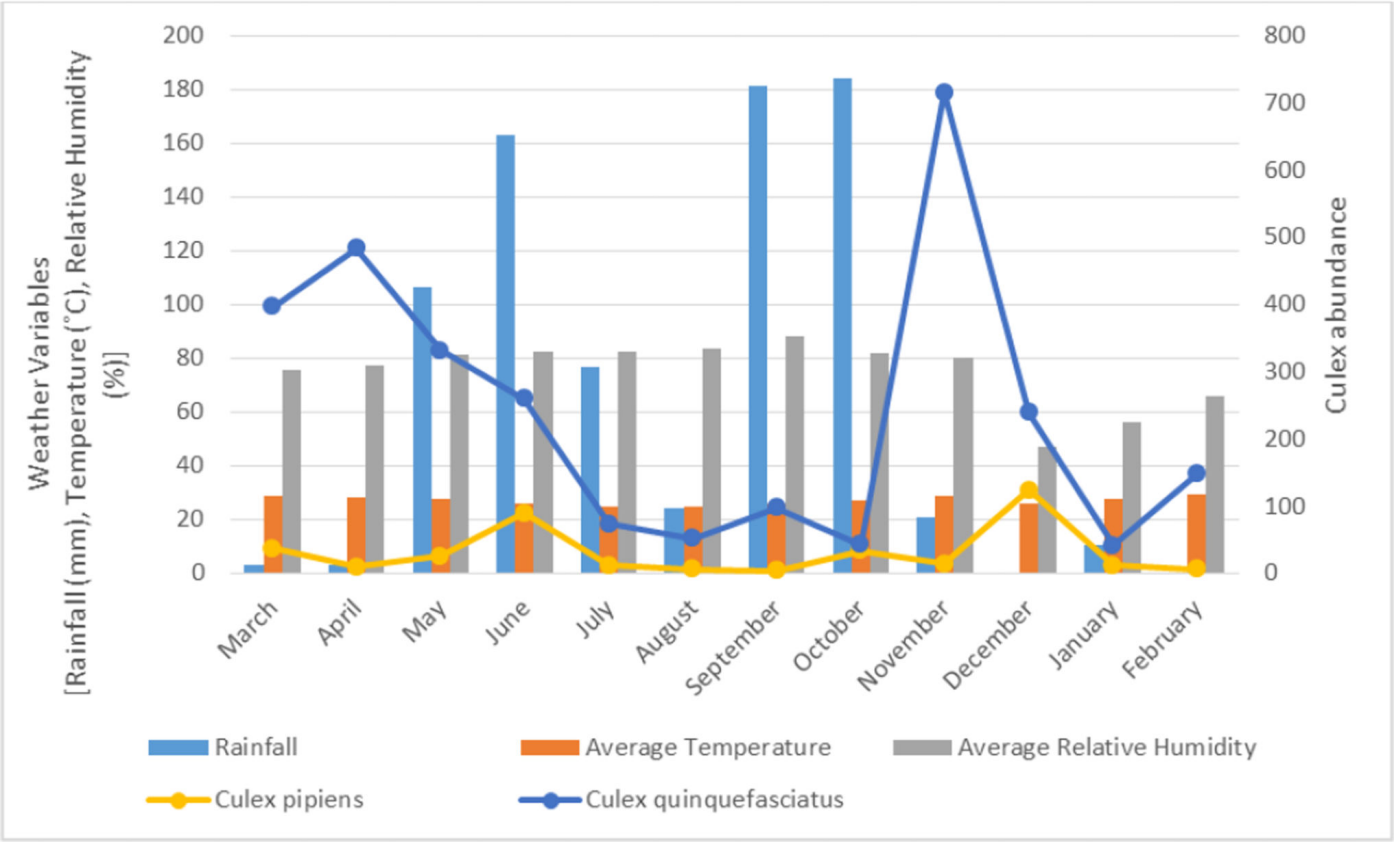
Two most occurring *Culex* mosquitoes, amount of rainfall, temperature and relative humidity from March 2015 to February 2016

*Culex quinquefasciatus* and *Cx. pipiens* were the two most occurring *Culex* mosquitoes, population of *Cx. quinquefasciatus* was high at the beginning of the rains and at the end of heavy rains. *Cx. pipiens* was present throughout the study period at a relative low number (Figure 4).

**Figure 5:**
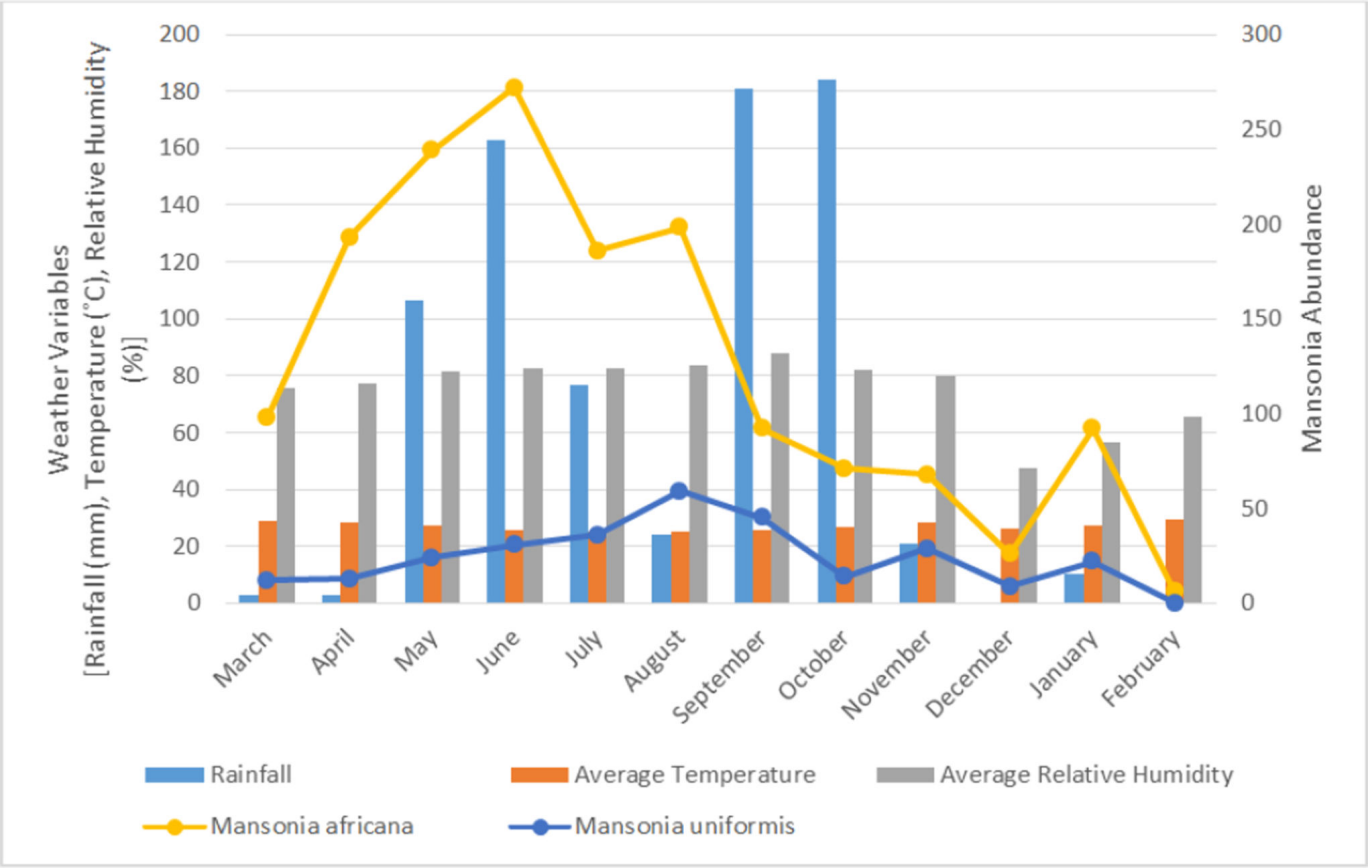
Two most occurring *Mansonia* mosquitoes, amount of rainfall, temperature and relative humidity from March 2015 to February 2016

*Mansonia africana* and *Ma. uniformis* were the two most occurring *Mansonia* mosquitoes, population of *Ma. africana* was high during the rainy season. *Mansonia uniformis* was present throughout the study period in good numbers (Figure 5).

## 3 Discussion

Sixteen mosquito species were encountered in this study out of which *Aedes aegypti, Ae. africanus, Ae. albopictus, Ae. mcintoshi*, *Culex pipiens*, *Cx quinquefasciatus*, *Mansonia Africana* and *Ma uniformis* are of significant medical importance. In addition, high vector population was found in periods succeeding months with high rainfall quantities. Activities of arboviruses have been previously documented in the study sites with the detection of Abadina virus in 1967 and 1969 from *Culicoides* and *Aedes fowleri* respectively, Rift Valley fever virus was isolated from *Culicoides* and *Cx. antennatus* in 1970, West Nile Virus from liver and spleen samples of rodents in 1969, Shamonda virus was isolated from cow blood sample in 1969 and 1970 and Sabo virus from *culicoides* in 1970 (University of Ibadan arbovirus research project, 1970). In essence many of the mosquito species collected in this study are important vectors of arboviruses. It is worthy of note that large outbreaks of yellow fever have occurred in Nigeria in the past (Carey et al., 1972; Monath, 1973; Fagbami et al., 1976; Nasisdi et al., 1989). *Aedes aegypti*, *Ae. albopictus* and *Ae. africanus* are potential vectors for yellow fever virus, and have been captured following previous yellow fever epidemics in some states in Nigeria (Lee and Moore, 1972; Onyido et al., 2009). These vectors can also transmit Dengue, Zika and other viruses. Evidence of other important viruses like Dengue and Zika viruses have also been previously documented in Nigeria (Carey et al., 1971; Fagbami, 1979; Fagbami, 1977; Fagbami et al., 1977). Other mosquito vectors of health significance encountered during the study included *Ae. mcintoshi*, *Cx. quinquefasciatus*, *Ma. uniformis* and *Ma. africana*, which are vectors for Rift Valley fever virus in some east African countries (Sang et al., 2010). *Culex quinquefasciatus*, a notable vector of West Nile virus was the most prevalent species encountered during this study. The presence of these vectors in farm environments implies that an outbreak can occur if a vireamic animal is introduced to this region. Similarly, a disease outbreak can occur if a vireamic wild reservoir is attracted to these farms.

Mosquito populations varied during the study period in response to prevailing weather conditions. This is expected since insect vectors are poikilothermic and subject to the fluctuating effects of abiotic factors in their environment of which weather is significant (Shope, 1991). The interplay of weather especially rainfall which provides the aquatic environment for larval development and also facilitates egg hatching for species that lays in containers and rely on flooding to trigger hatching is very important. Increased rainfall promotes development of favourable habitats for developing stages of insect vectors particularly the larval stage (Patz et al., 2003), thus favouring population growth (Gubler et al., 2001). Amount of precipitation in an area can also affect the availability of mosquito breeding sites. In our study, the highest abundance of mosquitoes did not occur in the month of June, September and October with the highest amount of rainfall. This may be due to the effect of excess rainfalls resulting into flooding that could wash off mosquito larval habitats before their complete development, thereby having a negative effect on local mosquito populations (Epstein, 2004). A similar finding was reported in the study of Uttah et al. (2013) in Imo state, Nigeria, where a decline in mosquito abundance was found in the months with the highest rainfall during the first year of their study. However, the highest abundance of mosquito was recorded during the month with highest rainfall in the second year of their study. Nevertheless, a sharp rise in mosquito population was recorded in the months succeeding the months with high rainfall, and this may be the result of a balanced interplay of abiotic factors, since flooding of larval habitats has ceased and mosquitoes have opportunity to complete their development. Similar spikes in the mosquito populations have been recorded in many countries in periods following abundant rainfall and are usually associated with disease outbreaks, for example Rift Valley fever outbreaks in Kenya (Davies et al., 1985; Himeidan et al., 2014). Different mosquito species responded differently to this effect of rainfall as seen in Figure 3, Figure 4 and Figure 5. However natural populations *Ae. mcintoshi*, a major vector of Rift Valley fever virus and *Cx. quinquefasciatus*, a vector of West Nile virus followed this pattern. Periods following months with high amount of rainfall represent seasons of highest risk for arbovirus transmission in our study sites. *Mansonia africana* responded differently to the effect of excessive rainfall because its larvae attaches to the roots of aquatic plants to obtain oxygen, therefore the effect of flooding was not pronounced on its population thus making it abundant in the rainy season. For these species the rainy season represent the period of highest risk for arbovirus transmission.

Temperature is also a very important factor in the dynamics of mosquito populations. Increase in environmental temperature has been observed to causes a decrease in mosquito generation time, longevity and life expectancy and increases the growth rate of vector populations, as well as decreasing the extrinsic incubation period and increasing the length of the pathogen transmission period (Patz et al., 2003). In this study temperature fluctuations recorded during the study period was not significantly correlated with mosquito abundance. Higher humidity increase mosquito survival (Gubler et al., 2001), decrease in humidity can adversely affect mosquitoes since they desiccate easily; survival rates decrease in dry conditions (Patz et al., 2003). Fluctuations in relative humidity during the study period were not significantly correlated with mosquito abundance except for *Mansonia* mosquito in which population increased as relative humidity did. Overall, the different mosquito genera responded differently to weather variables, their response may be related to their individual lifecycle or the duration of sampling, which was only 5 hours of 24 hours in a day.

## 4 Conclusions

In this study, we have shown the diversity and relative abundance of different mosquito species capable of propagating arboviral infections in livestock farms in Ibadan, southwestern Nigeria. The abundance of different mosquito vectors varied based on prevailing climatic conditions. An increase in mosquito population was recorded in months following heavy rainfalls. Overall, the abundance and diverse population of mosquito in two livestock farms are described. There is also a need to start intensive vector control efforts. Further studies aimed at detecting arboviruses from mosquitoes captured in these farms is ongoing in our laboratory.

## Figures and Tables

**Table 1:** List of mosquitoes collected from the University of Ibadan dairy farm and Teaching and Research farm from March 2015 to February 2016

Mosquito species	Number	Relative abundance (%)
*Aedes aegypti* (Linneaus)	211	3.41
*Aedes africanus* (Theobald)	9	0.15
*Aedes albopictus* (Skuse)	109	1.76
*Aedes mcintoshi* (Huang)	453	7.31
*Aedes metallicus* (Edwards)	37	0.60
*Aedes simpsoni* (Theobald)	28	0.45
*Aedes vittatus* (Bigot)	50	0.81
*Aedes sp.*	36	0.58
**Total *Aedes***	**933**	**15.07**
*Anopheles funestus* (Giles)	7	0.11
*Anopheles gambiae* (Giles)	10	0.16
**Total *Anopheles***	**17**	**0.27**
*Culex decens* (Theobald)	28	0.45
*Culex nebulosus* (Theobald)	69	1.11
*Culex pipiens* (Linneaus)	374	6.04
*Culex quinquefasciatus* (Say)	2 880	46.49
*Culex sp.*	45	0.73
**Total *Culex***	**3 396**	**54.82**
*Mansonia africana* (Theobald)	1 541	24.87
*Mansonia uniformis* (Theobald)	294	4.75
**Total *Mansonia***	**1 835**	**29.62**
*Uranotaenia annulata* (Leicester)	9	0.15
*Uranotaenia sp.*	5	0.08
**Total *Uranotaenia***	**14**	**0.23**
**Total Mosquito Species**	**6 195**	**100**

## References

[R1] Baba MM, Logue CH, Oderinde B, Abdulmaleek H, Williams J, Marcello A, D’agaro P, Hewson R (2012). Arbovirus co-infections in suspected febrile malaria and typhoid patients: A worrisome situation in Nigeria. Journal of Antivirals and Antiretrovirals Supplement.

[R2] Baba MM, Logue CH, Oderinde B, Abdulmaleek H, Williams J, Lewis J, Laws TR, Hewson R, Marcello A, D’agaro P (2013). Evidence of arbovirus co-infection in suspected febrile malaria and typhoid patients in Nigeria. The Journal of Infection in Developing Countries.

[R3] Boorman JPT, Service MW (1960). Some records of mosquitoes (Culicidae: Diptera) from the Niger Delta area. Southern Nigeria, West Africa Medical Journal.

[R4] Carey DE, Causey OR, Reddy S, Cooke AR (1971). Dengue viruses from febrile patients in Nigeria. Lancet.

[R5] Carey DE, Kemp GE, Troup JM, White JA, Smith EA, Addy RF, Fom LMD, Pifer J, Jones EM, Brls P, Shope RE (1972). Epidemiological aspects of the 1969 yellow fever epidemic in Nigeria. Bulletin of the World Health Organization.

[R6] Davies FG, Linthicum KJ, James AD (1985). Rainfall and epizootic Rift Valley fever. Bulletin of the World Health Organization.

[R7] Diallo D, Sall AA, Buenemann M, Chen R, Faye O, Diagne CT, Faye O, Ba Y, Dia I, Watts D, Weaver SC, Hanley KA, Diallo M (2012). Landscape ecology of Sylvatic Chikungunya Virus and mosquito vectors in south-eastern Senegal. PLoS Neglected Tropical Diseases.

[R8] Edwards FW (1941). Mosquitoes of the Ethiopian Region. III. Culicine Adults and Pupae. British Museum (Natural History).

[R9] Epstein PR (2004). Climate change and public health: emerging infectious diseases. Encyclopedia of Energy.

[R10] Fagbami AH (1979). Zika virus infections in Nigeria: virological and seroepidemiological investigations in Oyo State. The Journal of Hygiene.

[R11] Fagbami AH, Attah B, Fabiyi A, O’connor EH (1976). Yellow fever outbreak in South Eastern State of Nigeria--virological and serological studies. Nigerian Medical Journal.

[R12] Fagbami AH (1977). Epidemiological investigations on arbovirus infections at Igbo-Ora. Nigeria Tropical and Geographical Medicine.

[R13] Fagbami AH, Monath TP, Fabiyi A (1977). Dengue virus infections in Nigeria: a survey for antibodies in monkeys and humans. Transactions of the Royal Society of Tropical Medicine.

[R14] Gillies MT, De Meillon B (1968). The Anophelines of Africa South of the Sahara (Ethiopian Zoogeographical region).

[R15] Gubler DJ, Reiter P, Ebi KL, Yap W, Nasci R, Patz JA (2001). Climate variability and change in the United States: potential impacts on vector-and rodent-borne diseases. Environmental Health Perspective.

[R16] Harbach RE (1988). The mosquitoes of the subgenus *Culex* in southwestern Asia and Egypt (Diptera: Culicidae). Contributions of the American Entomological Institute.

[R17] Harbach RE, Howard TM (2007). Index of currently recognized mosquito species (Diptera: Culicidae). European Mosquito Bulletin.

[R18] Hayes E, Mackenzie J, Shope R, Heymann DL (2008). Arthropod-borne viral diseases. Control of Communicable Diseases Manual, 19th Edition.

[R19] Himeidan YE, Kweka EJ, Mahgoub MM, El Rayah EA, Ouma JO (2014). Recent outbreaks of Rift Valley fever in East Africa and the middle East. Frontiers in Public Health.

[R20] Jones B, Mckeever D, Grace D, Pfeiffer D, Mutua F, Njuki J, Mcdermott J, Rushton J, Said M, Ericksen P, Kock R, Alonso S, Grace D, Jones B (2011). Wildlife/domestic livestock interactions. Zoonoses (*Project 1*).

[R21] Karabatsos N (1985). International Catalogue of Arthropod-borne Viruses.

[R22] Kuno G, Chang GJ (2005). Biological Transmission of Arboviruses: New insights into components mechanisms, unique traits and their evolutionary trends. Clinical Microbiology Reviews.

[R23] Landsman WJ, Allan BF, Langerhans RB, Knight TM, Chase JM (2007). Inter-annual associations between precipitation and human incidence of West Nile virus in the United States. Vector-Borne Zoonotic.

[R24] Lee VH (1970). University of Ibadan Arbovirus Research Project. Annual Report.

[R25] Lee VH, Moore DL (1972). Vectors of the 1969 yellow fever epidemic on the Jos Plateau, Nigeria. Bulletin of the World Health Organization.

[R26] Mgbemena IC, Ebe T (2012). Distribution and occurrence of mosquito species in the municipal Areas of Imo state, Nigeria, Analele Universitatii din Oradea. Fascicula Biologie.

[R27] Monath TP, Wilson DC, Stroh G, Lee VH, Smith EA (1973). The 1970 yellow fever epidemic in Okwoga District, Benue Plateau State, Nigeria, I. Epidemiological observations. Bulletin of the World Health Organization.

[R28] Nasidi A, Monath TP, Decock K, Tomori O, Cordellier R, Olaleye OD, Harry TO, Adeniyi JA, Sorungbe AO, Ajose-coker AO, Van Der Laan G, Oyediran ABO (1989). Urban yellow fever epidemic in western Nigeria, 1987. Transactions of the Royal Society of Tropical Medicine and Hygiene.

[R29] Ogolo EO, Adeyemi B (2009). Variations and trends of some meteorological parameters at Ibadan, Nigeria. The Pacific Journal of Science and Technology.

[R30] Okoguna RA, Anosikeb C, Okereb N, Nwokeb EB (2005). Ecology of mosquitoes of midwestern Nigeria. Journal of Vector Borne Diseases.

[R31] Olaleye OD, Oladosu LA, Omilabu SA, Baba SS, Fagbami AH (1989). Complmenet-fixing antibodies against arboviruses at Lagos. Nigeria, Revue d’Elevage et de Medecine Veterinaire des Pays Tropicaux.

[R32] Olaleye OD, Tomori O, Schmitz H (1996). Rift Valley fever in Nigeria: infections in domestic animals. Revue Scientifique et Technique.

[R33] Olaleye OD, Tomori O, Ladipo MA, Schmitz H (1996). Rift Valley fever in Nigeria: infections in humans. Revue Scientifique et Technique.

[R34] Onoja AB, Adeniji JA, Olaleye OD (2016). High rate of unrecognized dengue virus infection in parts of the rainforest region of Nigeria. Acta Tropica.

[R35] Onyido AE, Ozumba NA, Ezike VI, Chukwuekezie OC, Nwosh EO, Nwaorgu OC, Ikpeze OO (2008). Mosquito fauna of a tropical museum and zoological garden complex. Animal Research International.

[R36] Onyido AE, Ezike VI, Ozumba NA, Nwankwo AC, Nwankwo EA (2009). Yellow fever vectors’ surveillance in three satellite communities of Enugu Municipality, Nigeria. Nigerian Journal of Parasitology.

[R37] Parrish CR, Holmes EC, Morens DM, Park EC, Burke DS, Calisher CH, Laughlin CA, Saif LJ, Daszak P (2008). Cross-species virus transmission and the emergence of new epidemic diseases. Microbiology and Molecular Biology Reviews.

[R38] Paulo MB, Renato A, Da Rocha TC, Eliane CG, Da Silva MAN, Walfrido KS, Joao N, Sueli GR, Jannifer OC, Pedro FV (2015). Serosurvey of arbovirus in free-living non-human primates (*Sapajus* spp.) in Brazil. International Journal of Environmental Analytical Chemistry.

[R39] Paz S (2015). Climate change impacts on west Nile virus transmission in a global context. Philosophical Transactions of the Royal Society of London, Series B, Biological Sciences.

[R40] Rajendran C (2000). Trends in mosquito control past, present and future. Recent Trends in Combating Mosquitoes.

[R41] Rogers D, Randolph S (2006). Climate change and vector-borne diseases. Advances in Parasitology.

[R42] Sang R, Kioko E, Lutomiah J, Warigia M, Ochieng C, O'guinn M, Lee JS, Koka H, Godsey M, Hoel D, Hanafi H, Miller B, Schnabel D, Breiman RF, Richardson J (2010). Rift Valley fever virus epidemic in Kenya, 2006/2007: the entomologic investigations. American Journal of Tropical Medicine and Hygiene.

[R43] Semenza JC, Menne B (2009). Climate change and infectious diseases in Europe. The Lancet Infectious Diseases.

[R44] Service MW (2003). Medical Entomology for Students.

[R45] Shope R (1991). Global climate change and infectious diseases. Environmental Health Perspectives.

[R46] Sule FW, Oluwayelu DO, Adedolun RAM, Rufai N, Mccracken F, Mansfield KL, Johnson N (2015). High seroprevalence of West Nile antibody observed in horses from southwestern Nigeria. Vector-borne and Zoonotic Diseases.

[R47] Tsai TF, Chandler LJ, Murray PR, Baron EJ, Jorgensen JH, Pfaller MA, Yolken RH (2003). Arboviruses. Manual of Clinical Microbiology.

[R48] (1970). University of ibadan arbovirus research project, Ibadan, Nigeria. Annual report.

[R49] Uttah EC, Wokem GN, Okonofua C (2013). The abundance and biting patterns of *Culex quinquefasciatus* Say (Culicidae) in the Coastal Region of Nigeria. ISRN Zoology.

[R50] World Health Organization (1975). Ecology and Control of Vector of Public Health Importance.

